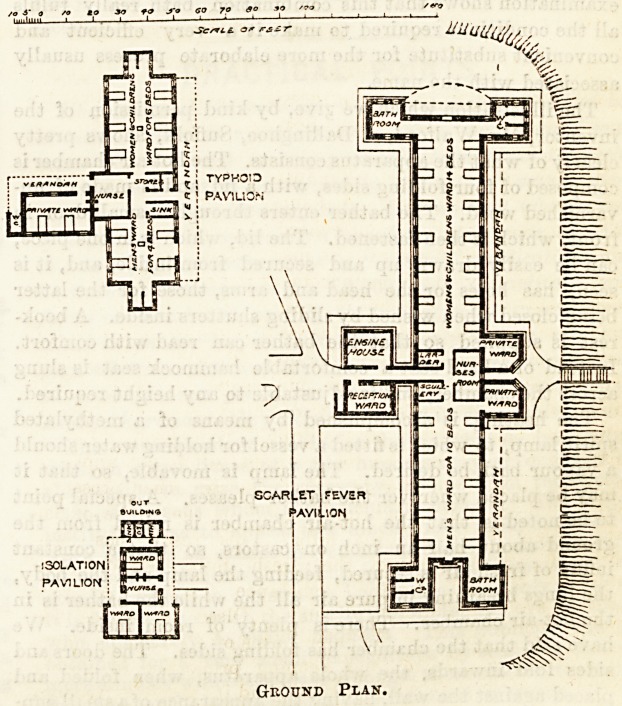# Hospital Construction

**Published:** 1894-12-01

**Authors:** 


### The Institutional Workshop.

### HOSPITAL CONSTRUCTION.

### THE BLACKBURN FEVER HOSPITAL.

This hospital, which is to serve as the isolation hospital for the County Borough of Blackburn, was opened by the Mayor in July last.

The site, which has an area of ten and a half acres,

Longshaw Lane.

Block Plan.

A, Administration Block. B, Scarlet Fever Pavilion. C, Typhoid Pavilion. D, Isolation Pavilion. E, Laundry. F, Lodge. G, OutBathing Place. H, Mortuary. I, Disinfecting and Stabling Block. K, Tennis Lawn.

is at Longshaw, on the outskirts of the town; it has an elevation at its eastern boundary of 575 feet above the mean sea level at Liverpool, and slopes gradually to the west.

The subsoil is clay and shale, which is well drained by a stream which runs through the site.

The buildings, which are all detached, are nine in number.

At the entrance to the ground are two small buildings, one being the porter's lodge the other the "outbathing place." This is in effect the discharge house for patients, and contains two waiting-rooms and a bath-room, the patient being supposed to undress in one room, pass from thence into the bath-room, and thence, by a second door, into the other waiting-room, where he assumes his disinfected clothing.

In the north-west corner of the site is an enclosed courtyard, having on one side the stable and ambulance house and on the other the disinfecting house. Just behind this latter is the mortuary, which consists of one room to serve the double purpose of mortuary and post-mortem room, and a narrow lobby.

The scarlet fever pavilion contains two large wards, one of fourteen beds for women and children, the other of ten beds for men. There are, in addition, two private wards for one bed each, one entered from the men's ward, the other from the women's ward. This arrangement, which was first carried out at Newcastle and has been copied in several other hospitals since, is a very bad one. For isolation purposes a ward opening out of another is quite useless; indeed, in any case, to make the sole means of access to a ward through another ward is a grave error. If these wards are intended for paying patients the arrangement is not a good one. A paying patient would be entitled to more privacy than these rooms would afford him, and might with reason object to having to go through the general ward in order to reach the bath-room or w.c. Between the two large wards is a long, narrow, and ill-lighted nurses' room a small scullery, and a larder. The one small window of the nurses' room looks into a narrow space between the two private wards. At the entrance to the pavilion is a reception ward and the engine-room in connection with the ventilating apparatus. The w.c.'s and bath-rooms are placed in towers projecting from the angles of the wards, and call for no remark except that, as so frequently occurs in hospitals of this kind, the accommodation for the cleansing and storing of bed-pans, &c., is sadly inadequate. The whole of this building is warmed and ventilated by mechanical means. The air which is filtered and, in cold weather, warmed by passing over coils of steam pipes, is extracted by means of two fans driven by steam. The air which is thus extracted passes along earthenware pipes to the laundry chimney, whence it escapes into the outer air. It is hoped that by this means the air will be effectually sterilised—whether this will be so in fact or not remains to be seen

Ground Plan.

but it may be as well to remark that the only possible test of sterilisation is a bacteriological one, and no mere record of temperature attained in the chimney, however high, can be accepted as conclusive evidence.

The typhoid pavilion is a T shaped building containing a ward for four men, one for six women and children, and a private ward for two beds. There does not appear to be any bath-room, and the private ward opens on to a verandah, which forms the sole means of access to the w.c.

In a small isolation pavilion, modelled on the lines of the puerperal pavilion of M. Tollet at the Lariboisiere Hospital, provision is made for three doubtful cases. There is a nurses' room, and access to the wards is by a verandah. The administration block contains the medical officers' rooms, and quarters for the matron, nurses, and servants. The kitchen offices are placed in a wing one storey in height.

The laundry building is placed at the extreme south of the site, and contains two distinct laundries, one being for the staff, the other for the patients.

The plans for the hospital were prepared in the office of the borough engineer (Mr. J. B. McCallum, C.E.), and that gentleman had the assistance of a former and the present medical officer of health for the borough. The result is, however, as far as we can judge from the plans, not as satisfactory as it might have been in small but important details.

## Figures and Tables

**Figure f1:**
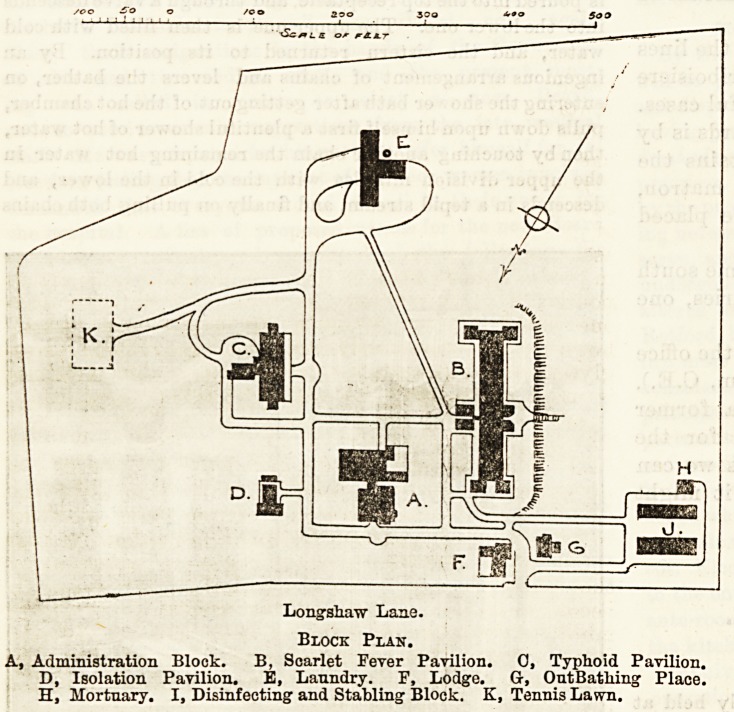


**Figure f2:**